# Cord Blood Banking Standards: Autologous Versus Altruistic

**DOI:** 10.3389/fmed.2015.00094

**Published:** 2016-01-08

**Authors:** Sue Armitage

**Affiliations:** ^1^MD Anderson Cord Blood Bank, Department of Stem Cell Transplantation, Division of Cancer Medicine, University of Texas MD Anderson Cancer Center, Houston, TX, USA

**Keywords:** cord blood, cord blood banking, standards, accreditation, regulations, autologous, altruistic, voluntary donations, autologous collections

## Abstract

Cord blood (CB) is either donated to public CB banks for use by any patient worldwide for whom it is a match or stored in a private bank for potential autologous or family use. It is a unique cell product that has potential for treating life-threatening diseases. The majority of CB products used today are for hematopoietic stem cell transplantation and are accessed from public banks. CB is still evolving as a hematopoietic stem cell source, developing as a source for cellular immunotherapy products, such as natural killer, dendritic, and T-cells, and fast emerging as a non-hematopoietic stem cell source in the field of regenerative medicine. This review explores the regulations, standards, and accreditation schemes that are currently available nationally and internationally for public and private CB banking. Currently, most of private banking is under regulated as compared to public banking. Regulations and standards were initially developed to address the public arena. Early responses from the medical field regarding private CB banking was that at the present time, because of insufficient scientific data to support autologous banking and given the difficulty of making an accurate estimate of the need for autologous transplantation, private storage of CB as “biological insurance” should be discouraged (1, 2, 3). To ensure success and the true realization of the full potential of CB, whether for autologous or allogeneic use, it is essential that each and every product provided for current and future treatments meets high-quality, international standards.

## Introduction

Following the first cord blood (CB) transplant for hematopoietic reconstitution (4), 27 years ago in France for a child with Fanconi Anemia, CB has moved rapidly from an experimental stem cell source to a widely accepted alternative to marrow as a source of hematopoietic stem cells. CB currently plays an important role in stem cell transplantation and cellular therapy, with an emerging role in regenerative medicine. CB transplantation is now a standard practice treatment option in both pediatric and adult transplantation for malignant and non-malignant disorders (5). CB has many advantages compared with other stem cell sources. From a safety perspective, it is a waste product; the collection process causes no harm to the donor infant and has a low risk of transmission of communicable diseases. Logistically, CB is an “off-the-shelf” product; it can extend transplant access to more racial/ethnic minority patients, currently underrepresented on donor registries worldwide and through the increased level of human leukocyte antigen (HLA) disparity that can be tolerated.

In the development of quality inventories, CB banks need to consider the critical issues for a high-quality CB unit to meet all current and potential clinical needs. The CB bank must take responsibility for providing first class products that are safe, pure, and potent. The CB unit being provided for clinical use may not only have been collected, tested, processed, and stored in a different country but also may have been banked over two decades ago. The quality of the CB product and its ultimate effect in the recipient’s treatment is reliant on both the procedures and processes at that time of cryopreservation and the subsequent maintenance of proper storage conditions. By accepting a CB unit for clinical use, the clinical program accepts full responsibility that the product has been correctly processed and tested. Regulatory and accreditation bodies, and registries have developed legislation, guidance, standards, and accreditation tools for CB banking. Compliance by the banks can provide clinical programs and the patients they treat the assurance that the CB unit not only has the correct HLA type and cell count but also contains viable hematopoietic progenitor cells that will result in durable engraftment or viable cellular immunotherapy products.

## Cord Blood Banking

A CB bank is a facility which stores umbilical CB for future use. Private and public CB banks have developed in response to this unique cell product with the potential for treating life-threatening diseases. There are also some, more recently emerging, hybrid CB banks providing both public and private storage.

(a) Public CB banks collect CB donations and store them indefinitely for possible clinical use. This unit is potentially available for any patient, if deemed a suitable HLA match. There is no charge to the donor, but the product is not stored specifically for that person or their family. These units are made available for search through national and international stem cell registries (6). Traditionally, public CB banking has been more widely accepted by the medical community (1, 2). In the United States (US), there are currently 17 public banks. Funding for public CB banks comes from a variety of sources, such as government, institutional, and philanthropic grants and donations. However, for most banks, the revenue received from the issue of units for clinical use is used to underwrite the costs of banking and additional collections. Without national support, public CB donation will never be an option for every potential donor.
(b) Private CB banks provide CB storage to families for potential future autologous or family use. For this service, the banks charge an up-front collection fee and then typically charge a yearly rate for ongoing storage of the CB unit. Contrary to public banks, family banks are not subject to many of the regulations and international accreditation; so, they apply less stringent quality criteria for the storage of CB units. In the US, there are currently 36 private banks.
(c) Directed donor programs are offered by a number of private and public CB banks. This type of banking is solely for sibling donor collection for those families who are likely to consider CB transplantation because a first-degree relative has been diagnosed with a disease that is treatable with allogeneic CB transplantation (7). The rationale for these programs is that if the new baby’s HLA type is compatible with the affected sibling, CB has the potential to be a good source of cells for transplant for the donor’s sibling. The likelihood that a sibling will share both HLA haplotypes is 25%.

Not all CB collections are viable for use: some do not contain sufficient cells, some may be contaminated, and others may have poor viability. Ultimately, a large percentage of the collections for public banking are not stored for clinical use – at the MD Anderson Public CB Bank, 70% of collections do not meet the required criteria for banking. However, many of these unsuitable units are made available for research and development. Quality standards in private banks do not necessarily follow the same strict criteria, which may result in stored units not being fit for use, if needed. Many private banks advertise possible future uses that are not entirely supported by clinical evidence, such as a cure for heart disease or autism or as a panacea. Many parents are led to believe they are buying biological insurance for their child and may feel that peace of mind afforded by private storage is worth the price. Unfortunately, they are often misinformed and misled by inaccurate information, vague promises, and aggressive marketing techniques that will exploit their feelings of guilt if they “miss this unique chance of saving their child’s life in the future” (3).

In the public CB banking arena, over 650,000 unrelated CB donations have been stored worldwide and listed on the Bone Marrow Donor’s Worldwide Registry (BMDW website: http://www.bmdw.org). In family CB banking, it in unknown how many CB units have been stored; two of the largest family banks in the US, both indicate on their websites that they each have ~500,000 units stored. More than 35,000 unrelated CB units have been distributed worldwide by public banks for allogeneic hematopoietic stem cell transplants, compared to <1,000 autologous transplants over the last two decades (8).

The role of a CB bank, public or private, is to assure patients and their families and their healthcare providers that they are committed to providing high-quality products that are potent, pure, and will not transfer an infectious, hematologic, or immunologic disease to the recipient. Regulations, standards, and accreditation in CB banking promote such assurance and continue to improve and progress the quality of the products for transplantation and cellular therapy treatments and develop regenerative medicine applications. It is paramount that all parties involved in collecting and supplying CB for clinical use work with the same goals and the same standards, including transplant outcome analysis as part of their quality management program.

## Cord Banking Regulations

Many countries now regulate CB products.

(a) In the US, the Food and Drug Administration^1^ regulates CB under the category of Human Cells, Tissues, and Cellular and Tissue Based-Products (HCT/P), in line with the 21 Code of the Federal Registry (9). Part 1271 and the Good Manufacturing Practices requirements in 21 CFR Parts 210 and 211. All private and public banks are required to comply with the FDA requirements for establishment registration and listing, current Good Tissue Practice regulations and donor screening and testing for infectious diseases (except when CB is used for the original donor). The FDA periodically inspects both types of banks. Public CB banks are also required to obtain a Biological License as the FDA considers unrelated allogeneic CB products to have a systemic effect, and therefore they regulate them as biological products and drugs. After October 20, 2011, every unrelated donor CB unit transplanted in the US must be either licensed or covered under an FDA-accepted Investigational New Drug (IND) (10). CB stored for personal use, for use in first- or second-degree relatives, does not require the agency’s approval before use.
(b) The European Union (EU) regulates CB on quality and safety issues related to donation, procurement, testing, processing, preservation, storage, and distribution of human tissues and cells through the EU Directives 2006/17/EC and 2006/86/EC (11). In 2004, the Council of Europe Committee of Ministers recommended that
  i. CB banking should be based on altruistic CB donation and used for allogeneic transplantation and related research;
  ii. promotion and the establishment of CB banks for autologous use should not be supported by member states or their health services;
  iii. where autologous CB banks are established, the promotional material or information provided to families must be accurate, and fully informed consent to CB storage must be obtained;
  iv. and autologous CB banks must meet the quality and safety standards set out in the Council of Europe’s Guide to safety and quality assurance for organs, tissues, and cells.
(c) Canada, Health Canada regulates CB under the Safety of Human Cells, Tissues and Organs for Transplantation, SOR/2007-118.
(d) The Australian Therapeutic Goods Administration (TGA) licenses CB through the Therapeutic Goods Act 1989 (12). Applying a risk management approach designed to ensure therapeutic goods supplied in Australia meet acceptable standards of quality, safety, and efficacy.

The similarity throughout these regulations is compliance with Good Manufacturing Practices to produce a safe and effective product for its intended use.

## Cord Blood Banking Standards

Cord blood standards are designed to provide minimum guidelines for all aspects of the CB bank’s operations, consistently assuring provision of quality products while protecting research, development, and new products. Standards are not intended to establish best practices or include all procedures that a CB bank should implement, if the standard of practice in applicable law establishes additional requirements. Compliance with standards is not an exclusive means of complying with the standard of care in the industry or with local, national, or international laws. It is critical that standards for CB banking be international, since CB products frequently cross international borders. The best CB unit, selected by HLA type and cell dose, is often located in another country. Transport across borders requires compliance with the regulations of the donor country and the receiving country. Both the AABB (formerly known as the American Association of Blood Banks) and the Foundation for the Accreditation of Cellular Therapy^2^ together with NetCord (NetCord-FACT) have developed international standards for CB banking quality management systems and technical requirements that both private and public banks may elect to follow for both unrelated and related products. The AABB standards for CB banks are incorporated into their cellular therapy standards and FACT-NetCord publish separate CB standards, aligned with their cellular therapy standards but unique to CB banking.

Standards are developed by consensus, based on the best available evidence-based science, with emphasis on research findings related to clinical outcome from CB recipients. When published data are not available, requirements are based upon accepted scientific theory. Standards are written by invited international leaders in cellular therapy programs and CB banks: knowledgeable clinicians, scientists, technologists, and quality experts that span the entire continuum of CB banking. Liaison representation from consumers and regulatory bodies are consulted throughout the development process to ensure detailed, comprehensive standards that address each aspect of a CB bank’s operations whether public or private:

(a) procurement of CB, regardless of technique or type of collection site;
(b) donor screening, testing, eligibility determination of the mother and donor infant, in accordance with applicable law;
(c) processing, cryopreservation and storage, including quarantine, testing and characterization of the product;
(d) making the product available for search, if unrelated;
(e) search and reservation process for selection of a specific unit;
(f) shipment of the CB unit, whether fresh or cryopreserved;
(g) release and distribution, including release testing to confirm product viability and hematopoietic potential.

The development process includes a period for public and professional review and comment, legal review, and approval by the relevant Board of Directors prior to publication of a new edition of the standards. The standards do not dictate how to meet a requirement and, even though they are international standards, it is not their intent to include every requirement of every government’s regulations. CB banking is a fast-evolving field and the standards need to evolve dynamically – both sets of standards are reviewed and revised on a defined periodic basis, with the option to publish interim standards based on changes to applicable laws and regulations, field experience, or misinterpretations.

In addition to the standards, an accreditation manual is developed simultaneously to provide guidance to applicants and to the on-site inspectors. This manual is intended to explain the intent and rationale for specific standards and to provide explanations, examples, and alternative approaches that may be helpful in the accreditation process. This is not an exhaustive list of possible ways to meet the standards, and the only intent is to provide examples since there are many effective mechanisms to achieve compliance with the standards and to inspect applicant CB Banks.

The NetCord-FACT Standards apply to CB units intended for unrelated use and to related units, collected, and stored for the directed use by a specific individual or family member of the donor infant. In previous editions, the standards distinguished between directed allogeneic and autologous units; since the ultimate use of a unit stored for family use is often unknown at time of storage, the concept of related replaces the terms directed allogeneic and autologous.

To be compliant with the both sets of standards, a CB bank is required to maintain a documented quality management program and use validated procedures and qualified supplies, reagents, and equipment. In addition, the NetCord-FACT standards require the tracking of clinical outcomes of patients receiving products from the bank.

## Accreditation

The basis for accreditation is to show documented compliance with the current edition of the standards. Accreditation establishes a uniform level of practice and promotes high-quality products/practices, leading to improved patient outcomes and elevates the bank’s position as a quality organization and informs patients, health insurance companies, and governments that your organization is dedicated to excellence in patient care and laboratory practices. Accreditation provides evidence of external validation through on-site inspections and facilitates the establishment of quality management and process control to minimize liabilities and regulatory non-compliance. Accredited organizations voluntarily seek and maintain accreditation through a rigorous process, demonstrating their belief that the patients’ needs are paramount.

All FACT-NetCord inspectors are expert volunteers, trained and active in the field; generally, three inspectors spend two full days at the facility to observe and review. Forty-six public CB banks and seven private CB banks are FACT-NetCord accredited (see footnote text 2), and a total of 69 CB banks, private and public, are AABB accredited,^3^ as of September 2015.

Any CB bank that lists their units on the NMDP registry are required to be either FACT-NetCord or AABB accredited. NetCord member banks are required to hold FACT accreditation. The Spanish National CB Program requires all CB units listed in the Spanish Registry REDMO to be accredited either by FACT-NetCord or by the Spanish CAT (Transfusion Accreditation Committee), responsible for inspecting blood banks in Spain. Several US states also require accreditation, including New York, New Jersey, and California. Any bank not accredited within those states is not legally permitted to collect CB from those states, even if the CB bank is based out of state.

## Conclusion

A public donation is made as a purely altruistic act, solely for the benefit of others. It has the potential to save the life of any person for whom the unit is a good match, including the person who donated it, if it is still available. Private CB banks store a unit solely for use by the donor or their family. However, the US, as in many countries, does not have an organized public CB banking system; in most of the country, public donation is not even possible – almost 90% of CB in the US are not collected. So for many people, the choice is not between public and private banking, it is between private banking and letting the CB go to waste.

Increasing evidence shows CB contains pluripotent stem cells that have the potential to differentiate into non-hematopoietic tissue, such as cardiac, neurologic, pancreatic, and skin tissue. Extensive laboratory research is taking place to explore the potential therapeutic benefit of CB under these circumstances. The results of this research will be necessary to formulate future recommendations regarding autologous CB banking.

Cord blood, whether donated for public banking or stored at the request of a family, is a unique cell product. To ensure success and the true realization of the full potential of CB now and in the future, whether for autologous or allogeneic use, it is essential that each and every product provided for current and future treatments meets high-quality defined international standards. The CB industry, private and public banks, stakeholders, and consumers must work together to develop thriving CB banking opportunities, producing high-quality CB products to improve outcome.

## Conflict of Interest Statement

The author declares that the research was conducted in the absence of any commercial or financial relationships that could be construed as a potential conflict of interest.

## Footnotes

^1^http://www.fda.gov/

^2^http://www.factwebsite.org/

^3^http://www.aabb.org/
